# Role of Endoscopy in Management of Upper Gastrointestinal Cancers

**DOI:** 10.3390/diseases11010003

**Published:** 2022-12-27

**Authors:** Jeff Liang, Yi Jiang, Yazan Abboud, Srinivas Gaddam

**Affiliations:** Karsh Division of Gastroenterology and Hepatology, Cedars-Sinai Medical Center, 8700 Beverly Blvd, ST, Suite 7705, Los Angeles, CA 90048, USA

**Keywords:** upper gastrointestinal cancer, endoscopy, cancer screening, pre-malignancy, gastroenterology, oncology

## Abstract

Upper gastrointestinal (GI) malignancy is a leading cause of cancer-related morbidity and mortality. Upper endoscopy has an established role in diagnosing and staging upper GI cancers, screening for pre-malignant lesions, and providing palliation in cases of advanced malignancy. New advances in endoscopic techniques and technology have improved diagnostic accuracy and increased the therapeutic potential of upper endoscopy. We aim to describe the different types of endoscopic technology used in cancer diagnosis, summarize the current guidelines for endoscopic diagnosis and treatment of malignant and pre-malignant lesions, and explore new potential roles for endoscopy in cancer therapy.

## 1. Introduction

Since the inception of the flexible endoscope in the 1950s, gastrointestinal (GI) endoscopy has played an ever-increasing role in the diagnosis of upper GI tract cancers and screening of pre-cancerous lesions [1]. Modern advances in endoscopic technology and technique have led to significant improvements in diagnostic accuracy and expanded the role of endoscopy in cancer staging and treatment [2,3]. 

**Figure 1:**
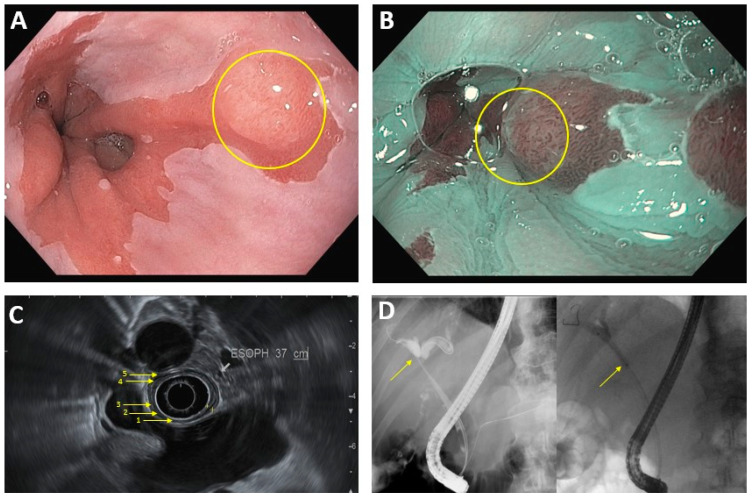
Common endoscopic modalities. (**A**): Conventional HD endoscopy showing Barrett’s esophagus with lesion (circled). (**B**): Image in 1A under NBI. Area of abnormal vascular and mucosal patterns (circled) is concerning for malignancy. (**C**): EUS of the esophagus. Arrows denote 5 alternating hyperechoic/hypoechoic layers: (1) mucosal surface, (2) muscularis mucosa, (3) submucosa, (4) muscularis propia, (5) serosa. (**D**): ERCP in hilar cholangiocarcinoma. Left image: dilation of intrahepatic bile ducts because of complete blockage of the common hepatic duct (arrow). Right image: improved flow of bile/contrast after deployment of plastic biliary stent across the malignant obstruction.

Conventional video endoscopy is the gold standard for diagnosing a wide range of upper GI malignancies. Chromoendoscopy, the intra-procedural application of stains and dyes to highlight abnormal mucosa, has traditionally been used to supplement video endoscopy and improve diagnostic yield [4]. Advances in HD imaging have led to the development of high-resolution endoscopy, allowing for closer magnification and clearer visualization of the mucosa [5]. Additionally, techniques such as narrow band imaging (NBI), which use camera filters or filter algorithms to identify malignant lesions, function as a modern “computerized virtual chromoendoscopy” that have lower interobserver variability compared to traditional chromoendoscopy while possibly providing similar (if not better) diagnostic accuracy (Figure 1) [3]. Autofluorescence imaging, which relies on detecting differences in light absorption/emission between dysplastic and normal tissue, is another form of image-enhancement endoscopy undergoing significant research, although its current utility is limited by a high false-positive rate [3,6]. Confocal laser endomicroscopy and endocytoscopy are newer, promising technologies that aim to visualize the mucosa at the cellular level in real-time, and additional studies are needed to better determine their optimal clinical utility [2,3,4,7,8]. 

In addition to direct visualization of luminal masses, evaluation of suspicious lesions in the gastrointestinal wall and extraluminal structures such as the pancreas can be performed via endoscopic ultrasound (EUS), and direct access to the biliary or pancreatic ducts for biopsy and palliative stent placement can be performed using endoscopic retrograde cholangiopancreatography (ERCP) [9,10,11,12]. Techniques such as fine-needle aspiration (FNA) can be used in conjunction with EUS to improve diagnostic accuracy, and technology such as elastography and contrast-enhanced ultrasound have been shown to improve sensitivity, specificity, and diagnostic yield when used to complement EUS-FNA [9,13,14]. Additionally, the function of EUS in visualizing and sampling the gastrointestinal wall layers, peri-intestinal lymph nodes, and surrounding structures gives it a crucial role in locoregional staging of malignancies [9,13,14]. Finally, there is an ever-growing therapeutic role for EUS, from supplementing ERCP in stent placement for malignant obstruction, to delivering anti-tumor agents via fine needle injection (FNI) in pancreatic cancer [15].

This article aims to explore the diagnostic and therapeutic function of endoscopy in several subtypes of upper GI cancers, and provides a brief review of new developments.

## 2. Management of Pre-Malignant Lesions

### 2.1. Barrett’s Esophagus

Barrett’s esophagus (BE), defined as >1 cm of columnar epithelium with intestinal metaplasia in the distal esophagus, is a known risk factor for development of esophageal adenocarcinoma (EAC) [16]. Screening is performed with the goal of risk stratification—presence of high-grade dysplasia with BE carries a 7% annual risk of progression to EAC. However, there is limited evidence for identifying patients who would most benefit from screening [16,17]. The most recent guidelines from the American College of Gastroenterology (ACG) recommend screening in men who experience GERD symptoms chronically (longer than five years) and frequently (more than once per week), and have two or more additional risk factors for BE/EAC (see Table 1) [16]. Screening in women is performed on a case-by-case basis, due to the relatively lower incidence of BE in women when adjusted for the common risk factors. Conventional upper endoscopy is considered the gold standard for BE screening. Recent studies show that unsedated transnasal endoscopy (uTNE) has similar sensitivity and specificity for detecting BE and is an acceptable, cost-effective alternative [16,18]. Additionally, new non-endoscopic methods, such as the Cytosponge-trefoil factor 3, are considered reasonable screening options. This device is a capsule attached to a string which can be swallowed, used to collect esophageal brushing samples, and withdrawn to evaluate for cell cytology and biomarkers [19]. While these new techniques have the potential to provide widespread, cost-efficient BE screening, patients found to have evidence of BE on uTNE or non-endoscopic methods will require conventional endoscopy for definitive treatment. Patients with no evidence of BE and absence of esophagitis do not need to be screened again, since the incidence of BE on repeat scope is only 2.3% [16].

Patients with columnar epithelium seen on endoscopy require histological confirmation of intestinal metaplasia in order to diagnose BE. Additional lesions that appear nodular, ulcerated, or flat with irregular mucosal contours should be resected via endoscopic mucosa resection (EMR) or endoscopic submucosal dissection (ESD) for histologic evaluation [16,17]. These techniques have similar efficacy, and can be curative for lesions that do not extend into the submucosa. If dysplasia is present on evaluation, subsequent endoscopic eradication therapy (EET) of residual BE is recommended; however, patients with low-grade dysplasia may elect for surveillance endoscopy with biopsy (performed every 6 months for one year, and yearly afterwards) [16,17]. Radiofrequency ablation (RFA) is the most commonly used modality for EET, although cryotherapy and argon plasma coagulation (APC) are reasonable alternatives [16,20,21,22]. Complete eradication of intestinal metaplasia (CEIM) is achieved at greater than 90% rate with combined EMR/ablative therapy [20,21]. Repeat endoscopy and biopsy is required to confirm CEIM, and multiple rounds of treatment are often necessary to achieve this goal [20,21].

**Figure 2:**
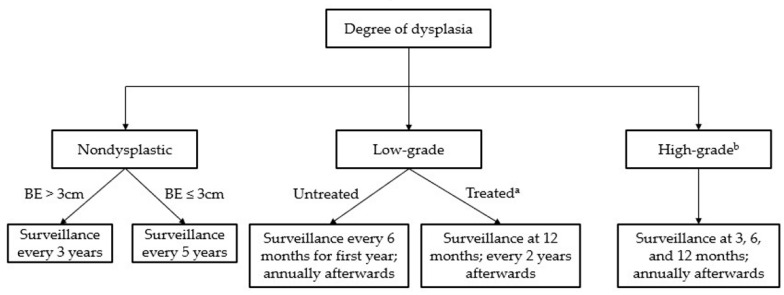
Surveillance guidelines for Barrett’s esophagus. ^a^ “Treated” assumes complete eradication of intestinal metaplasia (CEIM). ^b^ Treatment is recommended in all cases of high-grade dysplasia.

BE surveillance is performed with HD endoscopy, electronic chromoendoscopy (most commonly via narrow-band imaging [NBI]), and biopsy, and screening interval depends on degree of dysplasia (see Figure 2) [16,17]. Patients with nondysplastic BE do not require endoscopic therapy [16]. Some cases may be “indefinite for dysplasia” due to the presence of concomitant inflammation; in these instances, the ACG recommends a trial of acid-suppressive medications followed by repeat endoscopy after 6 months [16]. For patients with dysplasia who achieve CEIM after treatment, the annual incidence of recurrence for intestinal metaplasia is estimated to be between 8.6–10.5%, and the rate of recurrence for intestinal dysplasia is estimated to approximately 2% [16]. Therefore, the current ACG guidelines recommend repeat endoscopy at 3, 6, and 12 months for cases of high-grade dysplasia with annual surveillance afterwards, and at 1 year and every 2 years afterwards for cases of low-grade dysplasia [16].

### 2.2. Esophageal Epidermoid Metaplasia

Esophageal epidermoid metaplasia (EEM) is a new entity that is histologically analogous to leukoplakia in the mouth [23]. It is generally seen in the proximal to mid-esophagus and is believed to be a precursor to esophageal squamous cell carcinoma (SCC), as next-generation sequencing has revealed mutations in genes such as TP53, PIK3CA, and EGFR in EEM samples [24]. EEM is diagnosed via endoscopy, appearing visually as well-demarcated, white, cobblestone patches and histologically with hyperorthokeratosis and hypergranulosis [23,25]. While there are no guideline-directed treatments for these lesions, the current standard of care in our hospital is endoscopic mucosal resection or ablation. There are currently no guidelines on surveillance intervals for EEM; although, oral leukoplakia is generally managed with routine surveillance, esophageal lesions are not as easily monitored. Additionally, there are no known risk factors that precipitate EEM (unlike Barrett’s esophagus, which is associated with GERD); therefore, the clinical benefit of post-treatment follow-up is unknown [25].

### 2.3. Gastric Intestinal Metaplasia

Gastric intestinal metaplasia (GIM) is a transitional stage in the linear progression from gastritis to gastric carcinoma [26,27]. Endoscopic assessment of the extent and histological subtype of GIM aids in predicting the risk of progression to gastric cancer [26,27]. Incomplete intestinal metaplasia (colonic subtype on histology) is associated with a higher risk of progression to gastric cancer compared to complete intestinal metaplasia (small bowel subtype), and involvement of the gastric body and antrum is associated with a higher risk of progression than involvement of the antrum alone [26,27,28]. Evaluation for GIM is performed via biopsy of mucosal lesions and gastric mapping per the Sydney protocol [29], which involves taking biopsies from the greater and lesser curvatures of the body and antrum, and from the incisura [27]. Techniques such as narrow band imaging and chromoendoscopy have been shown to improve diagnostic yield compared to white light endoscopy alone [27,30,31,32].

Screening guidelines for pre-cancerous gastric lesions like GIM differ by country depending on the relative incidence of gastric cancer. In South Korea, the country with the highest age-standardized incidence of gastric cancer in the world, the National Cancer Screening Program (NCSP) offers screening with either endoscopy or upper-GI series every two years for all individuals between ages 40–75 [33]. Studies after implementation of the program have shown a significant reduction in the overall mortality rate of gastric cancer among individuals who underwent routine screening [33,34]. In contrast, the incidence of gastric cancer in the United States is low; thus, the American Gastroenterological Association (AGA) guidelines do not recommend screening for GIM [35]. While limited studies on Korean Americans suggest that immigrants from countries with a high incidence of gastric cancer are at higher risk of developing the disease, the AGA does not recommend different screening guidelines based on ethnicity at this time [36]. 

There is currently no treatment for GIM, although all patients with GIM should undergo *H. pylori* screening with subsequent treatment if they test positive [35]. While the current AGA guidelines recommend against routine surveillance, it states that individuals with increased risk for gastric cancer—such as incomplete intestinal metaplasia on histology, family history of gastric cancer, or immigration from a high incidence region—may elect to undergo surveillance endoscopy every 3–5 years [35]. As in the case of Barrett’s esophagus, any visible lesions should be resected via EMR or ESD. The AGA guidelines do not include recommendations for the management of gastric dysplasia, although the European Society for Gastrointestinal Endoscopy (ESGE) recommends repeat endoscopy at one year or six months for low-grade and high-grade dysplasia, respectively [35]. 

### 2.4. Pancreatic Cysts

Most pancreatic cysts are discovered incidentally on imaging, and are generally asymptomatic [37]. There are four major subtypes of pancreatic cysts—serous cystadenomas, mucinous cystic neoplasms (MCN), intraductal papillary mucinous neoplasms (IPMNs), and solid pseudopapillary neoplasms (SPN)—that differ in their malignant potential (Table 2) [37,38]. MRCP and MRI are the imaging modalities of choice for initial evaluation and distinguishing these subtypes. Clear cases of pancreatic pseudocysts or asymptomatic serous cystadenomas (microcystic appearance with central stellate scar on imaging) require no further evaluation. All SPNs should be evaluated for surgical resection [37]. 

However, cysts often cannot be categorized by imaging features alone, and IPMNs and MCNs frequently require additional evaluation to determine the risk of malignancy and guide further management [39,40]. Individuals with high-risk features—including cyst size ≥2 cm, solid cyst component, main pancreatic duct >5 mm in size, ≥3 mm cyst growth in a year, symptomatic cyst, obstructive jaundice, or family history of pancreatic cancer—should be further evaluated with EUS and FNA, which can assist in determining the histologic subtype of the cyst and assess malignancy risk [37]. A high level of CEA is seen in fluid studies of MCNs and IPMNs, and cytology can be performed to evaluate for dysplasia or carcinoma. However, fluid aspirate obtained with traditional FNA can have low cellular yield, limiting the diagnostic utility of fluid cytology [37]. Diagnostic yield can be improved with newer modalities such as through-the-needle tissue biopsy (TTNB), a technique that involves sampling the cyst using microforceps through a 19-gauge needle, or needle confocal laser endomicroscopy (nCLE) [41,42]. When the aforementioned techniques are used in conjunction with cross-sectional imaging and fluid studies, the diagnostic yield is approximately 93% [42]. Use of a 19-gauge needle is associated with a higher risk of post-procedure pancreatitis or intra-cystic hemorrhage compared to the standard 22-gauge FNA needle, so TTNB and nCLE are most beneficial in patients with nondiagnostic EUS-FNA results [41,42].

**Figure 3:**
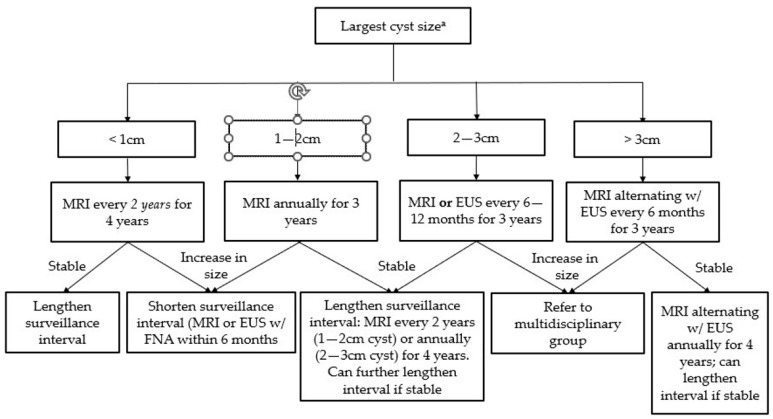
Surveillance guidelines for mucinous pancreatic cysts (Adapted from the 2018 ACG Clinical Guideline: Diagnosis and Management of Pancreatic Cysts). ^a^ Size-based surveillance guidelines only apply in the absence of high-risk features (see Table 1).

For IPMNs and MCNs without high-risk features, the surveillance interval depends on cyst size and growth (Figure 3) [39]. The duration of surveillance for IPMNs is controversial; the AGA guidelines suggest stopping surveillance after 5 years [37], although smaller studies have shown an increased risk of pancreatic cancer even beyond this time period [38]. MRCP is the preferred modality for surveillance, although EUS with FNA can be used in patients who cannot undergo MRCP [39,40]. The updated Fukuoka guidelines classify IPMNs as “high-risk” if there is main pancreatic duct (MPD) dilation >10 mm, obstructive jaundice, or a contrast-enhancing mural nodule >5 mm, and recommend resection in surgically fit patients. Additionally, anyone with “worrisome” features, such as MPD dilation between 5–9 mm or abrupt changes in MPD diameter, cyst size ≥3 cm, contrast-enhancing cyst walls, lymphadenopathy, or elevated CA 19-9, should undergo surgical evaluation [37,38]. 

Treatment for MCNs and IPMNs with high-grade dysplasia or carcinoma is surgical resection; however, the risks and benefits of surgery should be evaluated on a case-by-case basis, since older patients with other comorbidities died more frequently of causes unrelated to their pancreatic neoplasm [37]. MCNs without evidence of malignancy do not require post-operative surveillance; however, all IPMNs are associated with concomitant pancreatic adenocarcinoma (PDAC) and/or a risk of progression to malignancy, so post-operative imaging surveillance should be performed at least twice yearly [38]. Patients who underwent surgical resection for SPNs should undergo yearly imaging for at least five years [39,43].

### 2.5. Duodenal Adenomas

Duodenal adenomas can be categorized by location as ampullary or non-ampullary [44]. Non-ampullary adenomas can be seen using a standard endoscope, while ampullary adenomas often require a side-viewing endoscope due to their location [44]. Endoscopic or surgical resection is the treatment of choice for duodenal adenomas; in cases of ampullary adenomas, ERCP should be performed to evaluate for growth into the biliary and pancreatic ducts, since greater than 1 cm extension into the ducts is a contraindication to endoscopic resection [44,45,46]. No definitive guidelines exist for screening or surveillance of these pre-malignant adenomas due to the rarity of duodenal carcinomas, although the ASGE suggests initial post-treatment surveillance after 1–6 months with subsequent follow-up every 3–12 months for 2 years [44,47]. 

However, individuals with familial adenomatous polyposis (FAP) have approximately a 5% lifetime risk of developing duodenal carcinoma and require regular surveillance [48,49]. FAP is diagnosed clinically if an individual has ≥100 colonic polyps, or has <100 colonic polyps and a family history of FAP [50]. Therefore, the ASGE recommends anyone with a duodenal adenoma to also undergo a colonoscopy to evaluate for FAP [50]. Recommendations for management of duodenal polyps in FAP are based on the Spigelman classification, which categorizes patients into stage 0-IV based on the severity of their polyposis [48,49,51]. Most experts recommend that early-stage polyps can be monitored endoscopically roughly every 4–5 years (Spigelman 0-I) or 3 years (Spigelman II), while patients with Spigelman stage III or IV should undergo surveillance roughly every 6–12 months [48,49,51,52]. Additionally, individuals who are stage IV have greater than 30% chance of progressing to carcinoma within 10 years; therefore, all patients who are stage IV or stage III with high grade dysplasia are advised to undergo surgical evaluation [47,49].

Screening and surveillance are performed endoscopically; the side-viewing endoscope and ERCP can be useful for visualizing the ampulla and evaluating for biliary and pancreatic duct involvement [44,52]. Additionally, EUS can also be used in patients with Spigelman stage III and IV polyposis to evaluate for depth of invasion [44,52]. Adenomas that are less than 3 cm, Spigelman stage 0-III without high grade dysplasia, and do not have significant involvement of the nearby ducts can be treated with endoscopic papillectomy [44,52]. This can be performed via endoscopic resection or with ablative techniques such as RFA; the modality is usually based on proceduralist preference [44,53]. Recent studies have shown that RFA is also effective for treating residual neoplasm in the biliary or pancreatic duct after papillectomy [54,55]. However, post-treatment recurrence rates are high, and patients must be followed-up with interval monitoring and may require multiple rounds of treatment or definitive surgical resection [52]. 

## 3. Diagnosis and Staging

### 3.1. Luminal Upper GI Cancer

EGD is indicated for patients with risk factors and symptoms concerning for upper GI malignancy, such as persistent dyspepsia, esophageal dysphagia or vomiting in patients older than 50 years, anorexia, weight loss, early satiety, and unexplained iron deficiency anemia [56]. Endoscopy with biopsy is required to definitively diagnose malignancy of the upper GI tract [57,58]. Newer studies that incorporate confocal light endomicroscopy have found a sensitivity and specificity of greater than 90%, suggesting a role for this technique in identifying targets to biopsy or delineating tumor margins prior to resection [59,60,61].

Tumors are most common staged according to the TNM (Tumor depth or size, Nodal metastasis, and Metastatic disease) system. This evaluation begins with CT imaging, since the presence of metastatic disease precludes curative tumor resection [58,62]. In patients without evidence of distant metastasis, EUS is the first-line method for T (tumor depth) and N (nodal metastasis) staging due to its minimally invasive nature (Table 3) [57,63,64,65]. It has been shown to be effective in distinguishing T1/T2 from T3/T4 cancer, and tends to be more sensitive for identifying advanced cancers (sensitivity greater than 90% for T3 and T4 cancers) than earlier stage ones (sensitivity approximately 81% for T1 and T2) [57,63,66,67]. However, the accuracy of EUS may be limited by inter-observer variability, proceduralist experience, and the anatomic location of the tumor [63,65]. Additionally, EUS cannot reliably distinguish T1a from T1b tumors [57,67,68,69,70]. This is an important distinction, because T1b tumors carry a significantly higher risk of nodal metastasis and are generally treated surgically, whereas T1a lesions may be managed with endoscopic resection [71,72]. In these instances, T-staging may require endoscopic resection, which has been shown to have better accuracy and lower inter-observer variability [63,68]. 

EUS can inform N-staging by visualization locoregional lymph nodes or by lymph node biopsy via EUS-FNA. Four characteristics of lymph nodes seen on EUS—size > 10 mm, round shape, sharp borders, and absence of central intranodal vessels—are traditionally associated with a higher likelihood of malignancy, although its accuracy is poor (approximately 70% in esophageal cancer, and as low as 30% in gastric cancer) [57,63]. Studies have shown that accounting for three additional features—number of lymph nodes (≥5), involvement of the celiac nodes, and advanced primary tumor (T3 or greater)—improves the diagnostic accuracy for esophageal cancer (86% when ≥ 3 out of 7 features are present) [57,63]. When EUS findings are equivocal, EUS-FNA can be used to sample lymph nodes for histologic evaluation. However, EUS-FNA can yield false-positive results if the lymph nodes are accessed through the tumor site, and additional studies are needed to establish the definitive role of EUS-FNA in cancer staging [57,63,65].

### 3.2. Pancreaticobiliary Cancer

Pancreatic cancer is most commonly diagnosed via initial CT imaging; equivocal imaging results can often be clarified with EUS-FNA, which has been shown to have sensitivity and specificity greater than 90% [73]. Studies comparing EUS-FNA to cross-sectional imaging found that the former is especially accurate for diagnosing smaller pancreatic masses (<2 cm) [15,73]. 

Cholangiocarcinoma is traditionally diagnosed with cross-sectional imaging (MRCP is preferred), although distal extrahepatic neoplasms may be diagnosed via EUS-FNA or endoscopic retrograde cholangiography (ERC). Fluoroscopy-guided shaped endobiliary biopsy (FSEB) is a newer technique that involves manually re-shaping the endoscopy forceps to permit easier access to a biliary stricture, and has been shown to have high sensitivity for diagnosing both proximal and distal biliary neoplasms [74]. Intraductal cholangiocarcinoma is not easily accessible, and is typically diagnosed via multi-phase contrast MRI [75].

Gallbladder cancer is a rare entity that is diagnosed incidentally after cholecystectomy in approximately 50% of cases [76]. Individuals with a gallbladder polyp >1 cm seen on transabdominal ultrasound (TUS) are advised to undergo surgical resection. For patients with additional risk factors—such as primary sclerosing cholangitis (PSC), age > 50, focal wall thickening, or Indian ethnicity—cholecystectomy is recommended for polyps >5 cm [77]. EUS can be helpful in clarifying the diagnosis in less definitive cases [78,79]. EUS is more sensitive and specific than TUS in distinguishing neoplastic from non-neoplastic lesions; additionally, EUS-FNA can be performed to directly sample gallbladder lesions, with a reported accuracy between 80–100% [80]. 

Staging of pancreaticobiliary cancers generally begins with cross-sectional imaging, similar to the case with intraluminal cancers [75,78,81]. Surgical resection and staging laparoscopy are the most accurate modalities for evaluating gallbladder cancer and cholangiocarcinoma, respectively [75,78]. Pancreatic cancer may be accurately staged via endoscopy and imaging [81]. Studies have shown that EUS has a sensitivity of up to 94% for T staging and 82% for N staging [81]. It has also been shown to have high sensitivity and specificity for detecting portal venous invasion, but has poor accuracy for diagnosing arterial involvement of the tumor. This is an important distinction, since vascular involvement in pancreaticobiliary cancer makes the tumor non-resectable [81]. Helical CT remains the first-line technique for evaluating the vasculature; when these findings are non-diagnostic, supplementing with EUS has been shown to predict non-resectability with >90% accuracy [81]. 

## 4. Treatment of Cancer

### 4.1. Esophageal SCC/Adenocarcinoma

**Figure 4:**
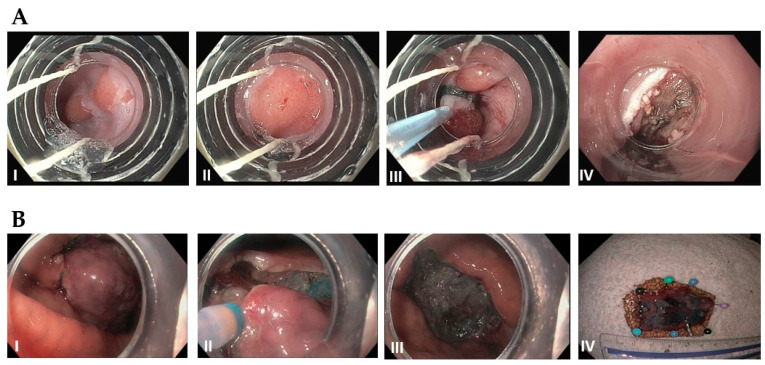
(**A**): Endoscopic mucosal resection (EMR) by band and snare technique: (I) Initial 1 cm, slightly raised esophageal lesion with surrounding Barrett’s esophagus. (II) Abnormal mucosal area is being capture completely into the cap with a cuff of normal surrounding mucosa. (III) Snare deployed around the band and resected using blended current. (IV) Resection base after cautery of underlying vessels. (**B**): Endoscopic Submucosal Dissection (ESD): (I) Initial large, well-demarcated lesion of the stomach. Lesions of the border are usually marked in the beginning and methylene bue or other lifting agents are injected to elevate the lesion (not performed here). (II) The lesion is resected with a needle-knife using electrocautery, starting at the previously demarcated margins. (III) Base of the lesion that has been fully resected. (IV) 3 cm resected lesion.

As mentioned above, stage T1a esophageal carcinoma in the absence of metastasis can be treated endoscopically, with the goal of achieving R0 resection (negative horizontal and vertical margins on histology) [71,82]. Studies have shown similar survival outcomes between endoscopic resection and esophagectomy, and lower morbidity, faster recovery, and decreased length of hospital stay with endoscopic treatment [83]. EMR has become the most common treatment technique for T1a esophageal cancer in the US. The ideal candidates are solitary, small (<1.5–2 cm), flat-type mucosal lesions without evidence of lymphovascular invasion [82,83,84]. While piecemeal resection may be performed for larger tumors, the excised samples cannot be accurately evaluated for negative margins [82]. ESD, is a newer technique that permits en bloc resection of larger tumors, and is associated with a lower risk of recurrence, but a higher rate of complications (Figure 4 for comparison of EMR and ESD technique) [82]. Endoscopic resection should always be followed up with ablative therapy of concomitant Barrett’s esophagus [83,84]. Patients who undergo curative resection still require interval surveillance, as the rate of developing metachronous tumors is high [84].

### 4.2. Gastric Cancer

Gastric adenocarcinoma makes up the overwhelming majority of gastric cancer cases, and most of the endoscopic treatment guidelines come from countries with a high prevalence, such as Japan and Korea. In general, well-differentiated tumors that are limited to the mucosa without ulceration, ≤2 cm without evidence of nodal or distant metastasis should be treated with EMR [84,85]. Well-differentiated non-ulcerated tumors >2 cm or ulcerated tumors ≤3 cm may be treated with ESD. Recent studies suggest that undifferentiated/poorly differentiated adenocarcinoma ≤2 cm are at low risk for developing nodal metastasis and can reasonably be treated with endoscopic resection [84]. However, undifferentiated adenocarcinomas often have indistinct borders that make achieving negative margins difficult, and may exhibit different behavior depending on histologic features (i.e., predominantly tubular vs. signet ring); therefore, additional studies are needed to specify which cases are most suitable for endoscopic treatment [84,86]. 

Patients found to have lympho-vascular invasion, positive vertical margins, or greater than 500μm tumor extension into the submucosa are at high rate for nodal metastasis and should be treated with gastrectomy [84,85]. In contrast, well-differentiated tumors with positive horizontal margins are at low risk for lymph node metastasis, and may be treated further with endoscopic resection [84,85]. Patients with negative tumor margins should undergo routine surveillance with endoscopy and CT imaging every 6–12 months [84,85]. 

In addition to adenocarcinoma, gastric neuroendocrine tumor (G-NET) is another type of gastric cancer that is being detected at increasing frequency [87,88,89]. G-NETs are traditionally classified into three types (gastric adenocarcinoma with neuroendocrine features on histology has been recognized as a fourth type in recent years), which guide management [87,88]. Type 1 comprises 70–80% of all G-NET cases, is caused by hypergastrinemia in the setting of chronic gastritis, and is generally superficial and sub-centimeter in size; in contrast, Type 2 G-NETs are less common, caused by hypergastrinemia related to Zollinger-Ellison Syndrome, and tend to be slightly larger in size [88]. The National Comprehensive Cancer Network (NCCN) guidelines recommend endoscopic treatment for Type 1 tumors that have not spread beyond the submucosa, and surgical resection of Type 2 G-NETs due to a higher risk for metastasis [88,89,90,91]. Type 3 G-NETs are sporadic, tend to be larger (>2 cm) on presentation, and have a >50% risk for metastasis; therefore, these are classically treated with gastrectomy and chemotherapy [87,88,89]. However, a small study from Kwon et al. showed that Type 3 G-NETs <2 cm without lymphovascular invasion may be safely treated with endoscopic resection [92]. The most recent NCCN guidelines recommend that localized, Type 3 G-NETs <1 cm can be resected endoscopically [91]. 

### 4.3. Gastrointestinal Stromal Tumor

Surgical resection was traditionally preferred over endoscopic therapy as the first line treatment for patients with resectable gastrointestinal stromal tumors (GIST) of the stomach, since these malignancies originate in the deeper muscularis propria layer [93]. However, advances in EMR technique have led to the development of endoscopic full-thickness resection (EFTR) [94]. This technique can be performed in an “exposed” manner, which involves dissection of the tumor followed by endoscopic closure of the serosa layer via Endoclip or suture (so the peritoneal cavity is briefly exposed to the intraluminal space), or via the “unexposed” approach by appositioning the serosal layers below the tumor prior to resection [94]. Submucosal lesions can also be accessed via the “tunneled” approach—the endoscope/resection device is passed into the submucosal layer, the tumor is removed, and the endoscope is withdrawn followed by closure of the tunneled tract [94]. A recent study comparing EFTR to laparoscopic surgery in treating small, focal GIST found complete tumor resection with no recurrence after 6 years in all cases, but EFTR was associated with a shorter procedure time and hospital length of stay [93]. Limitations of EFTR include reduced efficacy for lesions larger than 4–5 cm due to difficulty with closure, and inability to treat cases with metastatic lymphadenopathy [93,94]. Certain locations, such as the fornix of the gastric fundus, are also more challenging targets for endoscopic closure; however, techniques such as over-the-scope clip (OTSC) have been shown to be effective for supplementing EFTR in locations that are more difficult to access [95]. The role of EFTR will likely continue to grow with more advanced endoscopic technology and development of resection techniques.

### 4.4. Pancreatic and Ampullary Cancer

Pancreatic cysts are normally treated with surgical resection, and adenocarcinoma (PDAC) or neuroendocrine tumors (PNET) are treated with surgery (if resectable) or systemic chemoradiation. In recent years, endoscopic ablative procedures have been used with increasing frequency [96,97]. A small study by Park et al. examined the results of EUS-guided ethanol ablation in patients with small PNETs who were poor surgical candidates, and found that ≥60% of patients had a complete response after multiple treatments [98]. Similar levels of technical success and treatment efficacy were found when ethanol ablation followed by paclitaxel injection was used to treat pancreatic cysts [99]. RFA for non-resectable cancer has been shown to reduce chemotherapy requirement, and small studies have shown a good safety profile [97,100]; however, additional studies are needed to determine whether RFA provides any benefit to mortality or quality of life.

Similar to pancreatic cancer, resectable ampullary adenocarcinoma is typically treated surgically, and ampullectomy or pancreaticoduodenectomy currently remain the standard of care [44]. However, a few recent studies suggests that endoscopic papillectomy may be an appropriate treatment in cases of carcinoma in situ without intraductal extension [45,46,53]. Further investigation with a larger patient population and longer follow-up is warranted to determine the efficacy of endoscopic resection compared to surgery. 

### 4.5. Extrahepatic Cholangiocarcinoma

Endoscopic radiofrequency ablation (RFA) is a relatively new technique that promising outcomes in patients with malignant biliary obstruction who are not candidates for surgery [101]. When used with biliary stent placement, endoscopic RFA has been shown to prolong stent patency and may prolong survival [101]. Additionally, RFA may be effective for clearing occluded metal stents [101]. Adverse effects are rare, with cholecystitis being one of the most commonly reported [101]. RFA can also be applied in conjunction with local chemotherapy—a study by Yang et al. found that treatment with endoscopic RFA and local administration of 5-fluorouracil is associated with a median 5-month improvement in survival and a 1-month improvement in biliary stent patency compared to treatment with RFA alone (all patients in the study were receiving concomitant systemic chemotherapy) [102]. Using RFA with chemotherapy may produce a synergistic benefit by improving stent patency (thereby prolonging duration of localized chemotherapy delivery to the malignant stricture) and tumor sensitization [101,102]. Additional studies are needed to better evaluate the benefits and adverse effects of RFA and define its role in cancer therapy. 

## 5. Palliative Therapy

Patients with advanced upper gastrointestinal cancer are usually not candidates for curative therapy, and management of their condition is tailored toward palliative treatment [103]. Dysphagia, gastric outlet obstruction, malnutrition, abdominal pain, and obstructive jaundice are among the many complications that patients experience. Endoscopic techniques such as stenting, feeding tube placement, and celiac plexus block/neurolysis are commonly used palliative modalities due to their efficacy and minimally invasive nature. 

### 5.1. Stent Placement

Stenting is the standard of care for treating obstruction in the esophagus, gastric outlet, and pancreatic and biliary ducts. This procedure has a high rate of technical success with a short post-procedure length of hospital stay, and improves nutritional status, obstructive symptoms, and quality of life [104,105,106,107]. Acute post-procedure complications, such as rupture/perforation or pancreatitis (in cases of pancreatic stents) are rare; however, stent occlusion or migration frequently occurs after several months [104,107,108,109]. The incidence and average timing of these long-term complications depend on disease progression and the type of stent used [108,109,110,111].

The most common types of stents are self-expandable metal stents (SEMS) and plastic stents. SEMS are preferred in most cases due to a lower rate of complications [108,110], although plastic stents are easier to exchange and may be useful when the optimal drainage approach has not been determined [110]. The SEMS design can be covered (includes a fabric coating over a metal wire meshwork) or uncovered (wire meshwork only). Covered stents have lower rates of tumor ingrowth and occlusion compared to uncovered; however, covered stents are also associated with a higher rate of stent migration [110]. Due to these competing risks and benefits, the guidelines do not currently recommend one type of SEMS outside of certain indications (i.e., covered SEMS for sealing off tracheoesophageal fistulas) [107,110,111].

In cases of gastric outlet obstruction (GOO), surgical gastrojejunostomy can be performed in lieu of stenting. Surgery is associated with longer hospitalization and time to resume oral intake, but has fewer long-term complications requiring re-intervention [111]. In recent years, EUS-guided gastrojejunostomy is emerging as a feasible alternative to surgery [111,112]. This technique makes use of the innovative lumen apposing metal stent (LAMS) technology to form a fistulous tract between the stomach and jejunum. An international, multicenter study on 26 patients who underwent EUS-guided gastrojejunostomy showed a high (85%) rate of clinical success with minimal adverse effects, although further studies are needed to optimize this technique and determine its role in the management of GOO [112].

### 5.2. Enteral Feeding

Although patients with advanced upper gastrointestinal cancer are often malnourished, current guidelines recommend against routine nutritional support for cachectic patients with untreatable or advanced cancer due to the risk of procedure-related complications and lack of mortality benefit [113]. However, individuals who require nutritional support prior to receiving other life-prolonging treatments may benefit from enteral feeding via a nasogastric (NG) tube or the more permanent gastrostomy tube [114,115,116,117]. Additionally, enteral feeding tubes are reasonable in patients with head, neck or esophageal cancer, and may also be used for palliative decompression in patients with obstructive pancreatic cancer [118]. Optimizing nutritional status is associated with better post-operative cancer-specific survival in cases of resectable esophageal cancer, and more sessions of chemoradiation tolerated in patients undergoing multimodal therapy [116,117].

The most common techniques for enteral feeding tube placement are percutaneous endoscopic gastrostomy (PEG) or percutaneous radiographic gastrostomy (PRG); surgical feeding tube placement is less preferred because it is more invasive [118]. However, percutaneous approaches require safe anatomical access from the stomach to the abdominal wall and access with either an NG tube (PRG) or endoscope (PEG); if a segment of the colon is positioned ventral to the stomach or significant esophageal obstruction is present, surgical placement may be the only technically feasible option [114,118]. Studies comparing endoscopic vs. radiographic approaches have shown mixed results, and the preferred technique often depends on institutional resources and expertise [119,120,121,122].

Determining which patients would benefit from enteral feeding and the modality of tube placement should be an individualized decision that takes into account the patient’s nutritional status and prognosis, as well as technical procedural factors; therefore, the decision of feeding placement is best made after a multidisciplinary discussion [113,114,118].

### 5.3. Celiac Plexus Block

EUS-guided celiac plexus block (CPB) and neurolysis (CPN) are minimally invasive palliative modalities that can be used to manage malignancy-related pain, usually from pancreatic cancer, that is refractory to medical management [123]. CPB provides temporary pain relief via injection of an anesthetic and a steroid, while CPN permanently ablates of the celiac plexus with the injection of a sclerosant agent like alcohol or phenol [124]. A recent meta-analysis of 16 studies evaluating the role of EUS-guided CPN in the management of pain attributed to pancreatic cancer in 980 patients showed that 71% of them experienced pain relief [125]. Some studies also report that early CPN may reduce opioid use or delay dose escalation [126,127], although the findings have been mixed [128]. Additionally, EUS-guided CPN has not been shown to improve mortality, may be less efficacious when the cancer invades the celiac plexus, and can rarely result in serious complications such as paraplegia [127,129,130]. Further studies are needed to elucidate the role of CPN in the palliative management of pain associated with non-pancreatic upper gastrointestinal cancers.

## 6. Summary and Future Directions

The aforementioned techniques and technologic advances have transformed the care of patients with upper GI cancers. Advances in endoscopic visualization have led to earlier identification of pre-malignant lesions [30,131]. Additionally, procedures such as PEG tube and esophageal stent placement, and ERCP w/ biliary stenting give endoscopy a key role in palliative therapy [105,108,110,117]. Endoscopic diagnostic techniques have largely replaced the traditional surgical ones, leading to improved safety and cost-effectiveness. While surgeons in the past would need to perform exploratory laparotomy to diagnose, stage, and potentially resect a tumor, modern endoscopy and CT imaging provide a comprehensive pre-procedural mapping of the abdomen and GI tract, allowing today’s surgeons to take a patient to the OR with a specific goal for curative surgery.

Despite these advances, several limitations of upper endoscopy remain. The role of endoscopic therapeutic techniques is currently restricted to small, early-stage tumors and palliation. Tumor location may also precludes endoscopic treatment due to the risk of post-procedure structuring following EMR or ESD. Novel techniques, such as RFA in pancreatic neuroendocrine tumors or EFTR for resection of gastrointestinal stromal tumors, have shown promise in small studies but still need to be validated in larger clinical trials. Additionally, surveillance guidelines for gastric intestinal metaplasia and pancreatic cysts are limited by low quality of evidence. Further studies in these areas are needed to optimize cancer screening and preventative care for these patients.

### 6.1. AI/Deeping Learning and Endoscopy

There is a growing body of literature on deep learning in medicine in the past decade [132]. In regard to endoscopy, convolution neural networks (CNNs) have been used to develop computer vision software to assist endoscopists with detection and diagnosis of lesions [132,133]. Studies have shown that CNNs can identify pre-malignant lesions in Barrett esophagus, assist with detection of gastric polyps, and perform similarly to radiographic imaging in evaluating pancreatic cysts [134,135,136]. While deep learning’s definitive role in endoscopy has yet to be determined, it has the potential to considerably benefit the endoscopist by improving diagnostic accuracy, streamlining clinic workflow, and providing guidance for optimizing treatments [133].

### 6.2. Endoscopic Oncology

Advances in endoscopy have led to an ever-increasing role in cancer treatment. One prominent example of this is in the area of endoscopically guided injection of anti-tumor therapies. Endoscopic administration of 5-FU in late-stage esophageal cancer was reported in the 1990s, although clinical benefit was limited [137]. Since that time, developments in endoscopic techniques, such as EUS, have expanded the potential for extraluminal therapy and allow endoscopists to facilitate other treatments (i.e., EUS-guided brachytherapy seed placement) [138,139]. Advancements in chemotherapy delivery technology have improved the safety profile of intra-tumoral injections, and new cancer treatments have opened the door to a multitude of different injection-delivered treatment modalities [138]. Case reports and small-scale studies in patients with gastric cancer and pancreatic cancer have shown that intra-tumoral injections can be performed with a high degree of technical success and can shrink tumors without causing significant morbidity [138,140]. Additionally, clinical trials are underway examining the role of injection immunotherapy [138]. While further studies are needed to determine the clinical benefit of these procedures, the field of “endoscopic oncology” has demonstrated the potential to supplement existing treatments and address previously untreatable malignancies.

## 7. Conclusions

Advancements in endoscopy have provided highly accurate, non-invasive methods for detecting upper GI malignancy and pre-malignant lesions. Recent developments in endoscopic technique have made endoscopic treatments the preferred technique for resection of small focal lesions and palliative management of non-resectable malignancies, and ongoing studies offer the promise of an even greater role for the endoscopic oncologist.

## Figures and Tables

**Table 1:** Screening Guidelines for pre-malignant upper GI lesions.

	Screening/Surveillance Guidelines	High Risk Features	Screening or Surveillance Modalities
Barrett’s esophagus (BE)	Men w/ chronic GERD (>5 years) occurring > once/week and ≥2 risk factors (*ACG 2022*)	(1) Age > 50 (2) Caucasian race (3) Tobacco use (4) Obesity (5) First-degree relative with BE or EAC.	Upper endoscopy (EGD) -“gold standard” for diagnosis and treatment Transnasal endoscopy (screening only): -good sensitivity (91%) and specificity (96%) -cheaper than EGD -cannot perform interventions Cytosponge (screening only): -cheaper than EGD -cannot perform interventions -Newer technique; not widely used in the United States
Gastric intestinal metaplasia (GIM)	Routine screening NOT recommended. Surveillance every 3–5 years in patients with GIM and high-risk features *(AGA 2018)*	(1) Incomplete intestinal metaplasia (2) Extensive GIM (3) Family history of gastric cancer (4) Immigration from a high incidence region	Upper endoscopy
Pancreatic cystic neoplasms (IPMN and MCN)	Routine screening NOT recommended. Cysts with high-risk features should undergo EUS-FNA to evaluate histology. Cysts without high-risk features should undergo surveillance (*ACG 2018*).	(1) Cyst size ≥ 2 cm (2) Main pancreatic duct dilation > 5 mm * (3) Solid cystic component (4) Enhancing mural nodule > 5 mm * (5) ≥3 mm growth in 1 year (6) Obstructive jaundice * (7) Symptomatic cyst (8) Family history of pancreatic cancer (9) New onset diabetes	MRCP (preferred) -Generally preferred first-line EUS -Used in high-risk cases and when imaging is non-diagnostic
Duodenal adenoma	Patients with familial adenomatous polyposis: based on Spigelman classification (stage 0-IV). No definitive surveillance guidelines for patients without FAP.	Components of Spigelman class: (1) Increased polyp number (>20) (2) Polyp > 10 mm (3) Villous histology (4) High grade dysplasia	Upper endoscopy

* Individuals with main pancreatic duct dilation > 10 mm and other noted features should be referred for surgical evaluation.

**Table 2:** Categories of Pancreatic Cysts.

Cyst Category	Imaging Appearance (MRI and EUS)	Fluid Evaluation	Risk for Malignancy
Pseudocyst	Thick-walled Anechoic	Brown color Elevated amylase/lipase Low CEA	No
Serous cystadenoma	Microcystic with “honeycomb” appearance Central calcification	Thin, clear Low amylase/lipase Low CEA	No ^a^
Solid pseudopapillary neoplasm	Solid + cystic component	Necrotic debris	Yes
Mucinous Cystic Neoplasm (MCN)	Macrocystic +/− septations Peripheral calcifications +/− solid component ^b^	Mucinous Variable amylase (usually low) High CEA	Yes
Intraductal Papillary Mucinous Neoplasm (IPMN)	Dilated pancreatic duct ^c^ +/− septations +/− solid component	Mucinous High amylase High CEA	Yes ^d^

^a^ There are rare case reports of serous cystadenomas progressing to pancreatic cancer. ^b^ Solid component MCNs are at higher risk for malignancy. ^c^ Sub-categorized as Main Duct (MD)-IPMNs or Branch Duct (BD)-IPMNs. BD-IPMNs are the most common incidentally discovered pancreatic cyst. ^d^ MD-IPMNs have higher potential for malignancy than BD-IPMNs. Copyright and adapted from the ACG Clinical Guideline: Diagnosis and Management of Pancreatic Cysts, Table 2.

**Table 3:** Upper GI cancer Staging and Treatment.

	Staging Modalities	Endoscopic Treatment Options
Luminal Upper GI cancer ^a^	EUS: -First line for T-staging (sensitivity is 81% for stage T1/T2, >90% for T3/T4) and N-staging -EUS-FNA can help for N-staging via lymph node biopsy, although results can be technique-limited CT: -Used for M-staging -Lower sensitivity and specificity for N-staging, when compared to EUS	Endoscopic techniques (EMR, ESD) generally feasible for T1a tumors ≤2 cm Surgical resection vs systemic therapy for larger and more advanced tumors
Extraluminal upper GI cancer ^b^	EUS: -Sensitivity and specificity for T and N staging highest in pancreatic cancer (compared to gallbladder cancer or cholangiocarcinoma) Laparoscopy: -Most accurate diagnostic modality for gallbladder cancer and cholangiocarcinoma Cross-sectional imaging: -Modality of choice for diagnosing intrahepatic cholangiocarcinoma and evaluating resectability in pancreatic cancer -Used for M-staging	Generally, endoscopy has only a palliative role (RFA, stenting)

^a^ Luminal upper gastrointestinal malignancies include esophageal, gastric, and duodenal. ^b^ Extraluminal upper gastrointestinal malignancies include pancreatic, gallbladder, and cholangiocarcinoma.

## Data Availability

Data sharing not applicable. No new data were created or analyzed in this study. Data sharing is not applicable to this article.
